# Outcomes following repatriation from cardiothoracic intensive care to referring centres

**DOI:** 10.1186/2197-425X-3-S1-A160

**Published:** 2015-10-01

**Authors:** N Abu Al-Saad, J Burton, N Jones, S Webb

**Affiliations:** Papworth Hospital NHS Foundation Trust, Critical Care, Cambridge, United Kingdom

## Introduction

Appropriate repatriation of patients from specialist ICUs back to a referring unit is essential to maintain access to specialist services. Currently, this represents a small proportion of interhospital critical care transfers in the UK though trends towards increased regionalisation of specialist services is a potential driver for increases in such transfers.

## Objectives

To assess mortality and discharge destination of patients following transfer from Papworth Cardiothoracic Intensive Care Unit to referring hospitals. This includes patients receiving cardiothoracic surgery and those referred for Extracorporeal Membrane Oxygenation (ECMO).

## Methods

Retrospective analysis of Papworth ICU ICNARC data (Intensive Care National Audit and Research Centre) and ICU Computer Information System data from April 2012-April 2014.

## Results

169 patients were transferred from Papworth ICU to a general ICU during the study period. 46 (27.2%) were originally referred for ECMO, 123 (72.8%) in the non-ECMO group were predominantly elective & emergency cardiothoracic surgery patients.

The overall median Length of Stay (LoS) in Papworth ICU was 11 days (Interquartile range, IQR, 15.5 days). In the receiving hospital, median LoS was 18 days (IQR 28.5 days) for the ECMO group and 12 (IQR 28.5) days for the Non-ECMO group.

63% of patients survived to be discharged home from the receiving centre across both ECMO & non-ECMO groups. A further 13% and 11.4% respectively of patients were discharged to a rehabilitation facility (either short or long-term). 8 of 46 ECMO patients (16.7%) died following transfer from Papworth ICU to referring centre, 28 of 123 non-ECMO patients died after transfer (22.8%).

## Conclusions

The majority of patients referred for specialist cardiothoracic services over the study period, including ECMO, survived to be discharged home, despite representing a population of relatively prolonged ICU & hospital stay. Repatriation from our centre to referring general ICUs is generally safe & appropriate though there is likely scope for improvement in the efficiency and safety of such transfers. Future research in this area could assess methods for quality assurance and scope for standardisation.
